# Subcutaneous Bortezomib in Multiple Myeloma Patients Induces Similar Therapeutic Response Rates as Intravenous Application But It Does Not Reduce the Incidence of Peripheral Neuropathy

**DOI:** 10.1371/journal.pone.0123866

**Published:** 2015-04-14

**Authors:** Jiri Minarik, Petr Pavlicek, Ludek Pour, Tomas Pika, Vladimir Maisnar, Ivan Spicka, Jiri Jarkovsky, Marta Krejci, Jaroslav Bacovsky, Jakub Radocha, Jan Straub, Petr Kessler, Marek Wrobel, Lenka Walterova, Michal Sykora, Jarmila Obernauerova, Lucie Brozova, Evzen Gregora, Dagmar Adamova, Jaromir Gumulec, Zdenek Adam, Vlastimil Scudla, Roman Hajek, for the Czech Myeloma Group

**Affiliations:** 1 Department of Hemato-oncology, University Hospital Olomouc and Medical Faculty of Palacky University Olomouc, Olomouc, Czech Republic; 2 Department of Clinical Hematology, University Hospital Kralovske Vinohrady, Praha, Czech Republic; 3 Department of Internal Medicine, Hematology and Oncology, University Hospital Brno, Brno, Czech Republic; 4 Department of Clinical Hematology, University Hospital, Hradec Kralove, Czech Republic; 5 Department of Internal Medicine, University Hospital, Praha, Czech Republic; 6 Institute of Biostatistics and Analyses, Faculty of Medicine, Masaryk University, Brno, Czech Republic; 7 Department of Hematology and Transfusion, General Hospital, Pelhrimov, Czech Republic; 8 Department of Oncology, Hospital Novy Jicin, Novy Jicin, Czech Republic; 9 Department of Clinical Hematology, General Hospital Liberec, Liberec, Czech Republic; 10 Department of Clinical Hematology, General Hospital, Ceske Budejovice, Czech Republic; 11 Department of Hematology and Transfusion, Claudian Hospital, Mlada Boleslav, Czech Republic; 12 Department of Hematology and Transfusiology, Silesian Hospital, Opava, Czech Republic; 13 Department of Haematooncology, University Hospital Ostrava and the Faculty of Medicine, University of Ostrava, Ostrava, Czech Republic; Imperial College London, UNITED KINGDOM

## Abstract

**Objective:**

Subcutaneous (SC) application of bortezomib has been recently introduced as a new application route in multiple myeloma (MM) patients. We performed an analysis to compare the outcomes of bortezomib-based therapy in multiple myeloma (MM) patients treated using either intravenous (IV) or subcutaneous (SC) route of administration.

**Patients and methods:**

During January 2012 through December 2013, we performed a retrospective analysis of 446 patients with MM treated with bortezomib-based regimens (either once weekly – 63% or twice weekly – 27%) in both, the first line setting, and in relapse, with separate analysis of patients undergoing autologous stem cell transplantation. We assessed the response rates and toxicity profiles in both, IV and SC route of bortezomib administration.

**Results:**

The response rates in both IV and SC arm were similar with overall response rate 71.7% vs 70.7%, complete remissions in 13.9% vs 8.6%, very good partial remissions in 30.8% vs 34.5% and partial remissions in 27% vs 27.6%. The most frequent grade ≥3 toxicities were anemia, thrombocytopenia and neutropenia, with no significant differences between IV and SC group. There were no significant differences in the rate of peripheral neuropathy (PN). PN of any grade was present in 48% in the IV arm and in 41% in the SC arm. PN grade ≥2 was present in 20% vs 18% and PN grade ≥3 was present in 6% vs 4%.

**Conclusions:**

We conclude that subcutaneous application of bortezomib has similar therapeutic outcomes and toxicity profile as intravenous route of application. In our cohort there was no difference in the incidence of PN, suggesting that PN is dose dependent and might be reduced by lower intensity schemes rather than by the route of administration.

## Introduction

The introduction of bortezomib has significantly improved response rates and overall survival in patients with multiple myeloma (MM), and it has soon become the cornerstone of the treatment of both, relapsed as well as newly diagnosed MM [1–3]. One drawback of bortezomib-based treatment has been the necessity of intravenous application which is less convenient and might be difficult to ensure in patients with poor peripheral venous access.

Other routes of administration have not been approved until the results of the international randomized trial by Moreau *et al*., who confirmed similar efficacy and toxicity profiles of both intravenous (IV) and subcutaneous (SC) applications of bortezomib [4]. Previous phase I study in 24 patients showed similar systemic bortezomib exposure in both application routes with similar safety profile [5]. The later phase III study on 222 patients revealed that patients treated with SC bortezomib had similar therapeutic outc with similar toxicity profile but significantly lower incidence of peripheral neuropathy (PN) than in the arm with IV application (38% vs 53%) [4]. Based on the results of this study, SC application of bortezomib was approved by both FDA and EMEA in 2012.

Since then, many patients with bortezomib induced peripheral neuropathy have crossed to SC administration, and many new patients who initiated bortezomib based treatment have started with SC regimen in order to reduce the incidence and severity of peripheral neuropathy.

After two years of the use of SC bortezomib within the Czech Myeloma Group (CMG), we tried to compare the cohorts of patients with IV and SC administration in order to confirm the results observed in the international phase III study.

## Subjects and Methods

The retrospective analysis comprised of 446 MM patients treated with bortezomib-based regimens between January 2012 and December 2013 in the Czech Republic. All the patients were Caucasian, aged 18 years and older with measurable secretory MM.

The patients were treated with bortezomib either in induction or as the treatment for relapsed or refractory disease. We included all patients regardless of performance status, hematological, hepatic or renal function to prevent selection bias. Most patients were bortezomib-naïve, there were only 23 patients (8.8%) with bortezomib pretreatment with similar distribution in both, SC and IV arms. Intravenous injections of bortezomib were administered at concentration 1mg/mL as a 3-5s intravenous push, subcutaneous injections were administered at 2.5mg/mL in order to limit total volume.

The patients received 1.3mg/m^2^ dose of bortezomib with standard reduction scheme according to the reduction protocols in the case of adverse events. To reduce neurological toxicities, most of the patients received bortezomib once weekly instead of twice-weekly administrations. We excluded patients who switched from twice weekly to once weekly administration and those who had atypical bortezomib-based regimen (applications on day 1, 4, 8 and 15 in a 28-day cycle) as these would compromise the final results. In the rest 234 patients, 63% had bortezomib once weekly and 27% twice weekly.

Disease response was assessed using the uniform International Myeloma Working Group (IMWG) criteria [6]. Adverse events including the severity of peripheral neuropathy were assessed according to National Cancer Institute (NCI) common terminology criteria for adverse events (CTCAE, v3.0), and recorded in local documentation files [7]. All the acquired data were recorded in the Registry of Monoclonal Gammopathies (RMG) of the Czech Myeloma Group [8]. All participants provided written informed consent with inclusion of their data in the RMG and with their assessment. The records were strictly anonymized and de-identified prior to our analysis. The written consent was approved by the Ethical committee.

**Fig 1:**
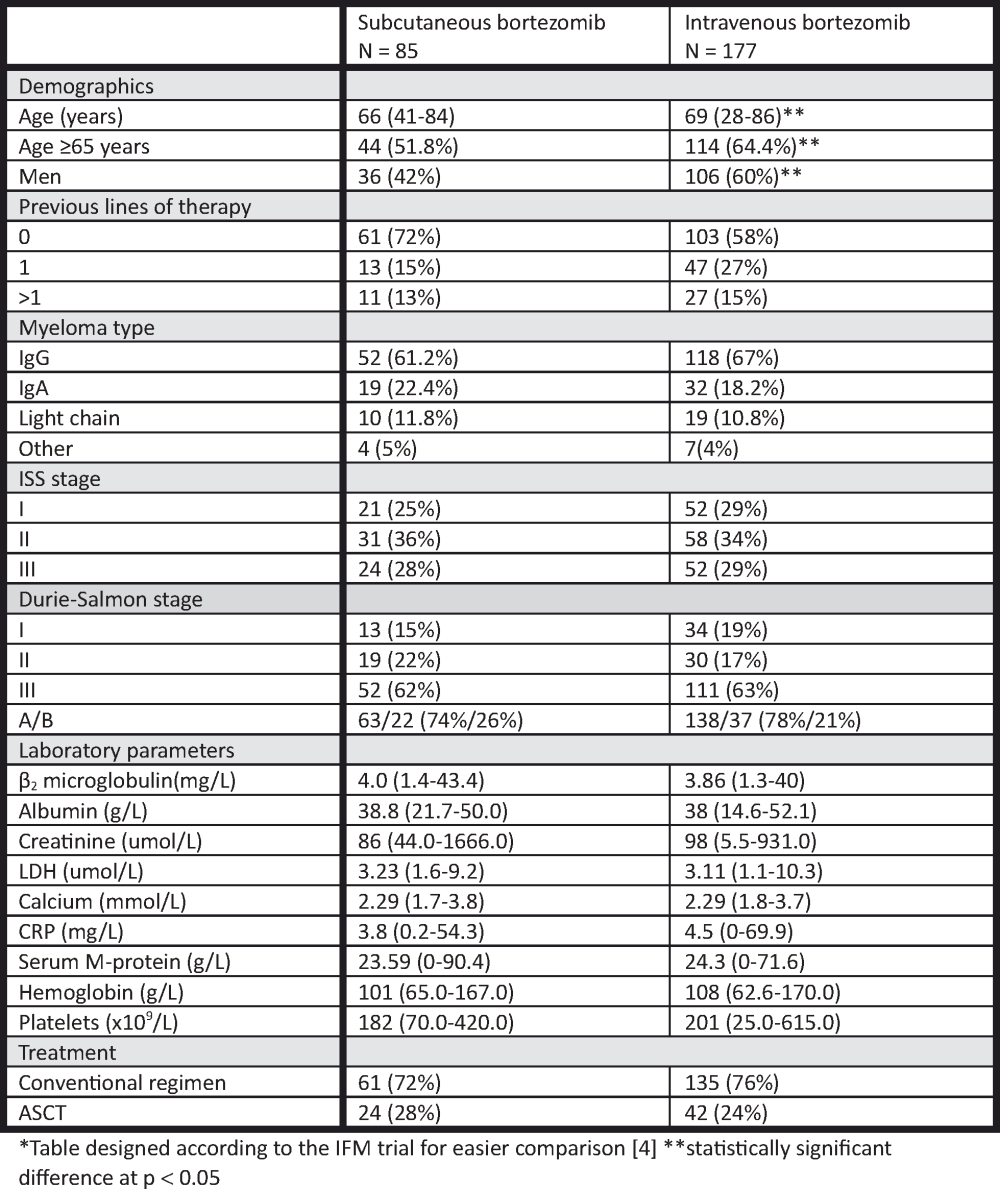
Patient demographics and baseline characteristics*. *Table designed according to the IFM trial for easier comparison [4] **statistically significant difference at p < 0.05.

From the whole cohort, we excluded patients who finished less than 4 cycles of treatment from other reasons than toxicity, patients who combined the regimen with other neurotoxic drugs or who switched to other treatment regimen, and patients varying on both IV and SC administration. All together 68% (177/262) patients were treated with IV and 32% (85/262) patients with SC bortezomib. Patients undergoing autologous stem cell transplantation (ASCT) following bortezomib-based induction (66/262) were assessed separately, with 64% (42/66) having SC bortezomib and 24 (36%) having IV bortezomib. Main characteristics of the groups of patients are in Fig 1.

For statistical estimation we used Mann-Whitney U test and Chi-square test at p < 0.05.

The study was approved by the Ethical committee of Medical Faculty of University Hospital Olomouc.

## Results

There were more men in the IV versus SC arm (59% vs 38%, p = 0.007) with slightly higher median age (71.3 vs 67.9 years, p = 0.024). The representation of monoclonal immunoglobulin types was similar in both arms as well as the representation of Durie-Salmon staging system (D-S) and International Scoring System (ISS). There was similar percentage of patients with renal insufficiency in both arms, too (21% vs 26%, p = 0.396). There were no significant differences between the arms regarding the level of serum M-protein (Median 24.3g/L vs 23.59g/L, p = 0.754), hemoglobin (Median 108.8g/L vs 106.2g/L, p = 0.237), thrombocyte count (Median 202.8x10^9^/L vs 189.1x10^9^/L, p = 0.168), serum calcium level (Median 2.3mmol/L vs 2.3mmol/L, p = 0.822), albumin (Median 36.8g/L vs 37.9g/L, p = 0.360), serum creatinine (Median 147.3umol/L vs 186.7umol/L, p = 0.614), beta-2-microglobulin (Median 6.1mg/L vs 6.6mg/L, p = 0.872), lactate dehydrogenase (Median 3.4umol/L vs 3.6umol/L, p = 0.345), and CRP (Median 9.9mg/L vs 7.0mg/L, p = 0.622), Fig 1.

The distribution of treatment lines was uneven in both arms but the difference was not statistically significant. There were 50% of patients undergoing bortezomib-based first line treatment in the IV arm (58% including ASCT group) versus 67% in the SC arm (72% including the ASCT group). Second line treatment was in 30% of patients in IV arm and in 15% of patients in SC arm, third line was in 11% in IV arm and in 8% in SC arm, and fourth and higher line was in 9% of patients in IV arm and in 10% of patients in SC arm.

The treatment regimens used within the IV and SC arms were following: CVD (cyclophosphamide, bortezomib, dexamethasone) in 58.2%/60.0%, VD (bortezomib, dexamethasone) in 10.7%/9.4%, BDD (bortezomib, doxorubicin, dexamethasone) in 9.6%/14.2%, VMP (bortezomib, melphalan, prednisone) in 6.0%/9.0%, bortezomib monotherapy in 1.1%/1.2%, BBD (bortezomib, bendamustine, dexamethasone) in 1.1%/2.4%, BP (bortezomib, prednisone) in 0%/1.6%, and other in 13.6%/2.4%,without significant difference (p = 0.069). The patients received median of 6.0 cycles in the IV arm versus 5.0 cycles in the SC arm (p = 0.014). The mean dose of drug for one administration 2.4mg vs 2.4mg, p = 0.416), total number of administrations (21.1 vs 20.5, p = 0.251) and total cumulative dose of bortezomib (50.2mg vs 47.7mg, p = 0.211) were similar in both arms.

There was no significant difference between the representation of bortezomib once weekly versus twice weekly in both arms (once weekly: IV arm—66.0%, SC arm—58.1%; twice weekly: IV arm 34.0%, SC arm 41.9%, p = 0.224). There were, however, significantly more patients with once weekly bortezomib administration in every single line of treatment (p ˂ 0.001).

**Fig 2:**
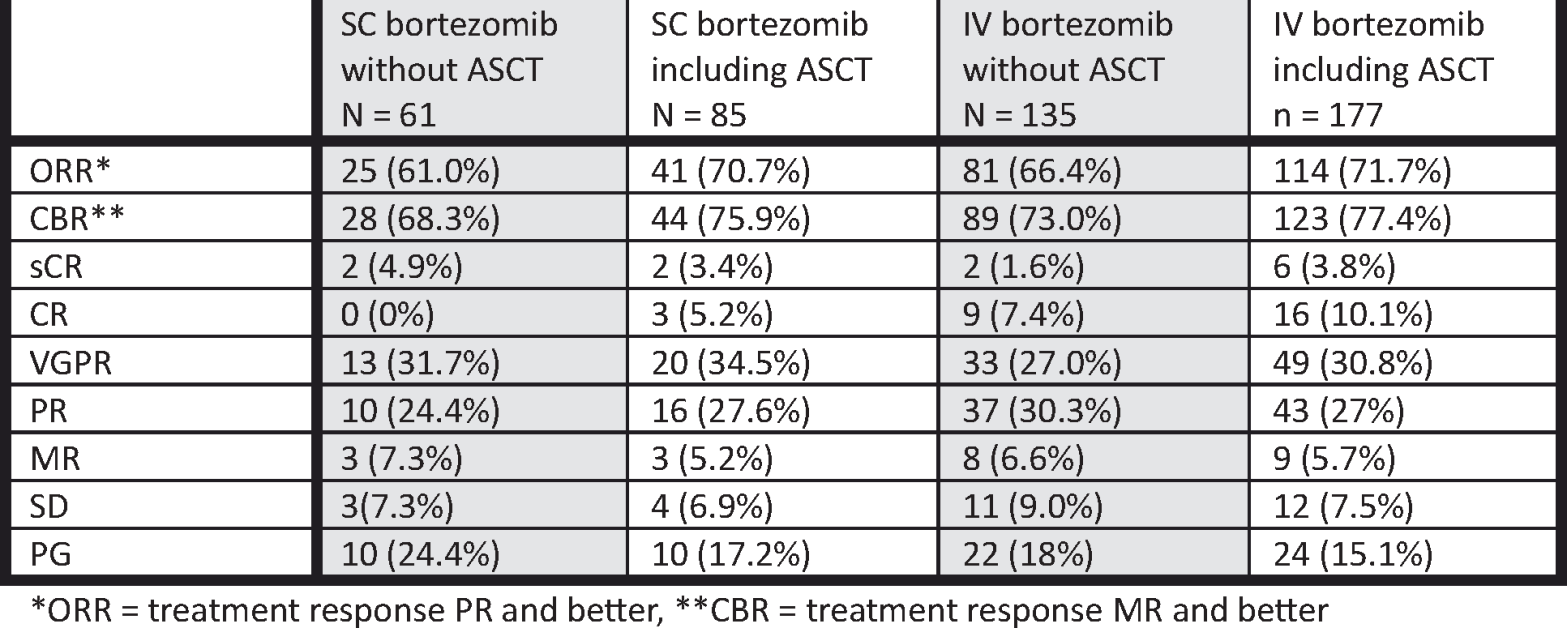
Rates of response to treatment by group in the response-evaluable population. *ORR = treatment response PR and better, **CBR = treatment response MR and better.

There was no significant difference in the treatment response in either IV or SC arm. The overall response rate in the conventional treatment cohort was similar (ORR 66.4% vs 61.0%, p = 0.261) as well as the rate of complete remissions (CR 8.9% vs 6.8%, p = 0.662), very good partial remissions (VGPR 28.6% vs 29.5%, p = 0.904), partial remissions (PR 50% vs 59.1%, p = 0.305) and minimal responses (MR 12.5% vs 4.5%, p = 0.114) with no significant difference. Inclusion of patients undergoing ASCT in both IV and SC arms increased the percentage of CR and VGPR (40.9% vs 39.7%, p = 0.609) and the ORR (71.7% vs 70.7%, p = 0.949) with no significant difference in either arm, Fig 2. We observed no statistically significant difference in treatment response between IV and SC arm when comparing subgroups of patients undergoing first line treatment or the treatment of relapse, either.

**Fig 3:**
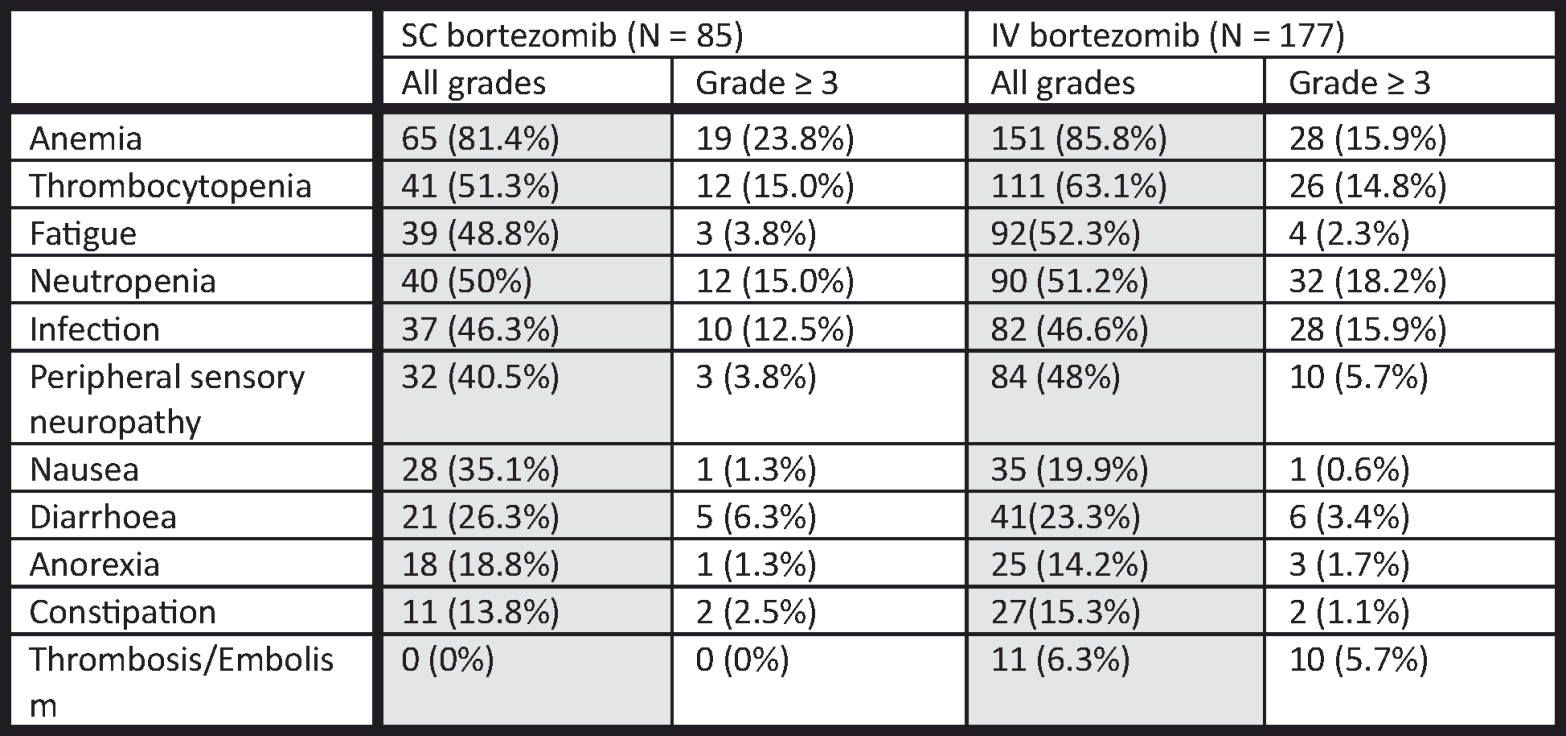
Rates of adverse events in SC and IV aplication routes of bortezomib.

Both the IV and SC arm registered similar toxicity profile (all toxicities 99% vs 96%, p = 0.569). There was no statistically significant difference in the incidence of grade 1, grade 2, and grade ≥3 anemia, thrombocytopenia, fatigue, neutropenia, infection, nausea and vomiting, anorexia, diarrhea and constipation, Fig 3. There were slightly more patients with grade ≥3 thrombembolism in the IV arm (5.7% vs 0%, p = 0.013).

There were 11 patients with preexisting neuropathy in the SC arm in comparison with 18 patients with preexisting neuropathy in the IV arm, without statistically significant difference (18% vs 13%, p = 0.073). The rate of neuropathy after treatment was similar throughout the whole cohort, regardless of IV or SC route of administration (total 48% vs 40.5%, grade 1–28% vs 22.8%, grade 2–14.3% vs 13.9%, grade ≥3–5.7 vs 3.8%, p = 0.782), Fig 3. We recorded no significant difference in dose reduction in either IV or SC route of administration (14.1% vs 9.4%, p = 0.271). There were 7 patients who interrupted the treatment due to toxicities, all of them being in the IV arm. The exact reason for treatment interruption, however, is not specified in the RMG [8].

## Discussion

Introduction of SC route for bortezomib application meant crucial step towards optimizing the use of bortezomib in MM patients [9]. The trial by Moreau *et al*. demonstrated equivalent efficacy of SC and IV administration with similar toxicity profiles in both groups, moreover, with significantly reduced rate of PN in patients with relapsing MM [4]. Similar efficacy together with toxicity profile of SC bortezomib in the setting of newly diagnosed MM has been recently demonstrated in both transplant eligible patients, and transplant ineligible, frail patients [10,11].

SC administration provides less invasive approach without the need for peripheral venous access or central venous devices, still with maintained efficacy, and, possibly with enhanced comfort for the patient [4,9,12,13]. The Czech Myeloma Group started with SC application soon after its approval by EMEA and local committees. During 2012–2013, practically all CMG bortezomib protocols changed into both IV and SC application versions with maintained dosing schedule. At this time we could follow approximately similar cohorts of MM patients treated either with IV or SC bortezomib. In order to confirm the results of the international randomized trial, we performed a large retrospective analysis comparing the groups of patients within IV and SC arms.

Our cohort, unlike the international randomized trial, included patients in the first line setting, and also patients with bortezomib-based induction followed by ASCT [4]. Due to shorter course of bortezomib treatment before ASCT, this cohort was assessed separately. As expected, the response rates were similar in both arms, showing non-inferiority of the SC arm. Our response rates were slightly better than in the trial by Moreau *et al*. (ORR in the IV arm 66% vs 42%, ORR in the SC arm 61% vs 42%) despite shorter median length of treatment, in part due to the inclusion of combined regimens (Moreau *et al*. used single agent bortezomib), and very likely because of more than half of patients being treated in the front line setting (62.5% of patients in frontline setting vs 37.5% in relapse).

The incidence of grade 3 or worse adverse events was in accord with the international randomized trial, the most common grade ≥3 being anemia (15.9% vs 23.8%), thrombocytopenia (14.8% vs 15.0%) and neutropenia (18.2% vs 15.0%), Fig 3. Several patients with SC bortezomib had local skin reaction (red non-itching skin exanthema surrounding the needle insertion) which was, however, grade I only and disappeared spontaneously. As the patients did not complain about the local reaction, most of them were not recorded in the database, and were therefore not included in the study results. There was a significant difference in the rate of grade 3 thrombotic events, probably due to limited number of patients and low frequency of the event (0% in the SC arm vs 5.6% in the IV arm).

Unlike the trial by Moreau *et al*., we report identical incidence of neuropathy of all grades in both IV and SC arms. In comparison with the international randomized trial, we report lower PN rates. Especially grade ≥2 and grade ≥3 PN in the IV arm was significantly lower in our cohort (grade ≥2–41% vs 20%, grade ≥3–16% vs 6%). There might be some difference in the incidence of ≥3 PN as there was a slightly higher percentage of patients with pre-existing neuropathy in the SC arm followed by insignificantly lower incidence of ≥3 PN. Still, the difference is far lower than in the international randomized trial. Bortezomib-induced PN is the major dose-limiting or even treatment limiting toxicity in MM patients [14]. The mechanism is still not fully understood but it is attributable to metabolic changes caused by bortezomib accumulation in the dorsal root ganglia cells leading to dysregulation of calcium homeostasis and to dysregulation of neurotrophins [15]. PN caused by bortezomib is predominantly small fibre, sensory and distal with only rare cases of motor nerve involvement [16]. Most cases of PN are dose-dependent, reversible, and improve after bortezomib is reduced or withheld [17–21]. Several recent papers have reported on favorable results with similar efficacy with lower incidence of PN in regimens with bortezomib-weekly administration [14,22]. Therefore we adopted bortezomib 1.3mg/m^2^ once weekly administration schedule in most of our patients, unlike the dosing in the international randomized trial which used bortezomib 1.3mg/m^2^ twice weekly based on Millenium prescribing information [16].

**Fig 4:**
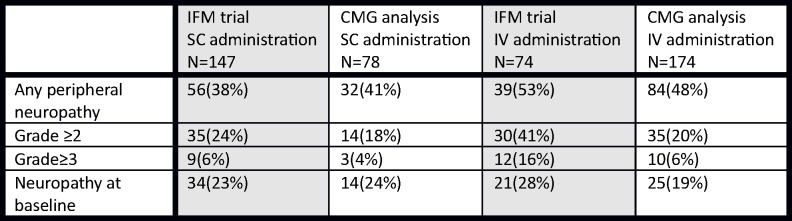
Comparison of the incidence of PN in patients treated with IV and SC bortezomib in the IFM trial and CMG analysis.

Weekly dosing of bortezomib in most of the patients (63%), together with shorter median course of the treatment and substantial number of patients being treated in the first line setting probably caused the difference between the rate of PN in our cohort in comparison with the trial by Moreau *et al*., Fig 4. Also, the presence of neuropathy at baseline was slightly lower in our cohort. We had slightly more patients with pre-existing neuropathy in the SC arm but the difference was not significant. In comparison, the international randomized trial had insignificantly more patients with pre-existing neuropathy in the IV arm. Nevertheless, we could trace significant incidence of PN in both arms without any differences between the IV and SC routes of bortezomib administration.

The reason for lower incidence of PN in the SC arm described by the international randomized trial is not yet fully understood. As the pharmacokinetic sub-study of the trial revealed identical area under the curve (AUC) representing equivalent systemic exposure of bortezomib in both SC and IV arms, there might be the reason in the first “peak” level of the drug (mean maximum plasma concentration) after administration as this was the only significant difference between IV and SC administration routes in the trial by Moreau *et al*. [4,23]. This phenomenon, however, does not explain the occurrence of PN usually after more cycles of chemotherapy which points to systemic exposure and cumulative dose rather than to the peak concentration of the drug. On the other hand, the updated results of the French study show significant differences in PN in SC and IV arm regardless of cumulative dose of bortezomib [24]. Our results oppose these findings as there we found no significant differences in PN despite similar total cumulative dose in both arms (with even insignificantly higher cumulative dose in the IV arm).

At this time, we could trace only two more retrospective studies presented at international congress that suggest that the route of bortezomib might influence the incidence of PN [25,26]. One of them studied PN in a small cohort of patients with MM and AL amyloidosis [25]. Other administration schedules than weekly SC bortezomib (SC twice a week, IV weekly, IV twice a week) caused significantly more PN. The cohort was, however, heterogeneous, with median of two bortezomib courses only, and the numbers of patients with PN in each administration regimen was limited. The latter study included 1058 MM patients and was well balanced despite significantly worse performance status and higher rate of general co-morbidities in the IV arm [26]. The results favored SC administration; however, they were aimed at time to dose reduction rather than objective assessment of the grade of PN. Still, we lack some more evidence that would support lower incidence of PN in patients with SC administration of bortezomib. Instead, the results of our analysis suggest that the incidence of PN is dose dependent and might be reduced by lower intensity schemes (weekly bortezomib) rather than by the route of administration. Nevertheless, we have confirmed that SC route of bortezomib administration is safe, comfortable and with similar efficacy as IV administration. As expected, there were no significant differences between the IV and SC application of bortezomib regarding therapeutic outcome and toxicities, the treatment was with high response rates and with fair tolerability. Despite high number of all toxicities (up to 99%) in both arms, most of them were grade 1–2 only and even those with grade ≥3 were predictable and manageable.

We acknowledge the possible bias of the retrospective assessment compared to a prospective randomized trial. Our cohort is different from the patients included in the trial of Moreau *et al*.—our cohort consists of patients on the time basis and with different treatment schedules, there are differences between the number of patients in each arm, moreover, we included patients regardless of treatment line whereas there were only relapsed patients in the international randomized trial. Further analyses are therefore required in order to confirm the contribution of SC bortezomib.

We conclude that SC administration of bortezomib in MM patients is safe, comfortable, and non-inferior when compared to IV route. The rate of PN in our study was, unlike the study by Moreau *et al*., similar in both SC and IV arms, suggesting that low-intensity bortezomib dosing regimens lead to the reduction of PN rather than the route of administration.

All relevant data are within the paper and its Supporting Information files.
